# The Role of Interleukins in Colorectal Cancer

**DOI:** 10.7150/ijbs.46651

**Published:** 2020-06-14

**Authors:** Jingjing Li, Ling Huang, Hanzhang Zhao, Yuheng Yan, Jing Lu

**Affiliations:** 1 Department of Clinical Medicine, Grade 2017, School of Basic Medical Sciences, Zhengzhou University, Zhengzhou 450001, Henan, China.; 2 Department of Pathophysiology, School of Basic Medical Sciences, Zhengzhou University, Zhengzhou 450001, Henan, China.; 3 Collaborative Innovation Center of Henan Province for Cancer Chemoprevention, Zhengzhou University, Zhengzhou 450001, Henan, China.; 4 State Key Laboratory of Esophageal Cancer Prevention and Treatment, Zhengzhou University, Zhengzhou 450001, Henan, China.

**Keywords:** Colorectal cancer, Interleukins, Molecular mechanism, Clinical therapy

## Abstract

Despite great progress has been made in treatment strategies, colorectal cancer (CRC) remains the predominant life-threatening malignancy with the feature of high morbidity and mortality. It has been widely acknowledged that the dysfunction of immune system, including aberrantly expressed cytokines, is strongly correlated with the pathogenesis and progression of colorectal cancer. As one of the most well-known cytokines that were discovered centuries ago, interleukins are now uncovering new insights into colorectal cancer therapy. Herein, we divide currently known interleukins into 6 families, including IL-1 family, IL-2 family, IL-6 family, IL-8 family, IL-10 family and IL-17 family. In addition, we comprehensively reviewed the oncogenic or antitumour function of each interleukin involved in CRC pathogenesis and progression by elucidating the underlying mechanisms. Furthermore, by providing interleukins-associated clinical trials, we have further driven the profound prospect of interleukins in the treatment of colorectal cancer.

## Introduction

Colorectal cancer (CRC) is the third most common cancer and the fourth leading cause of cancer-related death worldwide 1. Currently, the main treatment for CRC is surgical resection, supplemented by systemic chemotherapy and local pelvic radiotherapy 2. However, CRC treatments still face enormous challenges. In 2018, 1.8 million new cases were diagnosed, and one-third of these new patients had metastatic cancer 3. Although, in developed countries, the use of early screening has significantly increased the 5-year survival rate for patients with CRC 4, the latest data show that the prevalence of early-onset CRC is increasing, especially the incidence rate in young people with rectal cancer 3. The study also shows that the incidence rate of CRC in the United States is expected to increase by 90% by 2030. In such a tough situation, people are eager to seek new therapeutic strategies.

Interleukins (ILs) can be divided into several families with more than 40 subfamily members. They can interact with a variety of cells that alter the immune system and act on a wide range of cancers. In the past several years, ILs have attracted substantial attention because of their distinct roles in CRC (Table 1) that provide a new and promising strategy for CRC. In general, ILs facilitate CRC by promoting tumorigenesis 5, tumour growth 6, angiogenesis 7, and cancer cell invasion and metastasis 7 and inhibit CRC via complex pathways. In addition, some clinical trials in progress are expected to lead to a breakthrough in the treatment of CRC. In this review, we summarize the distinct roles of the diverse IL families with an impact in CRC and emphasize their internal mechanisms and clinical applications by focusing on recent studies in the hope of providing some useful inspiration for continued pursuit of yet unrevealed mechanisms and further clinical research.

## IL-1 Family

"IL-1" was first discovered in 1979 8. After a long period of research, it was found that the IL-1 family was comprised of IL-1α, IL-1β, IL-1Rα, IL-18, IL-33, IL-36α, IL -36β, IL-36γ, IL-36Rα, IL-37 and IL-38. IL-1α, IL-1β, IL-1Rα and IL-33 were found to be increased significantly in CRC, while IL-18 was decreased in colon cancer (CC) patients 9-11. IL-18Rα and IL-18Rβ are IL-18 receptors, and IL-18Rβ was overexpressed in rectal cancer. In mammalian CRC, ST2 was the upregulated receptor of IL-33. Furthermore, sST2, a soluble form of the IL-33 receptor, was downregulated in highly metastatic CRC cells compared with the level in low-metastatic CRC cells 10-12.

### Members with tumour-promoting effects

#### IL-1α

IL-1α could negatively change the chemosensitivity, which is of great value for the clinical treatment of CRC. A recent study used HCT116 colorectal carcinoma cells as the research objects to depict the details of IL-1α-dependent protein-protein interactions (PPIs). Specifically, these cells were also used to explain the experimental phenomena showing that exogenous IL-1α with 5-Fu changed the expression of cell adhesion molecules and that exogenous IL-1α promoted chemosensitivity in both chemosensitive and chemoresistant CRC cell lines, while endogenous IL-1α promoted chemosensitivity only in the chemosensitive HCT116 cells. The altered cell adhesion molecule suggested that the potential for enhanced cancer metastasis and 5-FU-induced cell death were increased with the treatment of exogenous IL-1α 13.

IL-1R1 and IL-1R2 are IL-1α receptors. Because of the pro-tumour function of IL-1α, blocking the IL-1R1 and IL-1R2 has attracted attention. Anakinra is an IL-1R antagonist. Experimental studies have shown that anakinra can reduce interstitial fluid pressure (IFP), which is measured indirectly by perfusable tissue fraction (PTF) and tumour blood flow (TBF). High levels of IFP in CRC metastasis may reduce the efficacy of antitumour drugs. The experiment showed that anakinra couldn't change the PTF or TBF, because of the experimental design and dearth of data samples, this result needs to be further studied 14. In contrast, TRAP IL-1, which is a soluble receptor, reduced the proliferation of CRC cells by inhibiting IL-1R1 15. Additionally, the single immunoglobulin IL-1 receptor-related molecule (SIGIRR) downregulated IL-1R signaling, whereas a SIGIRR isoform (SIGIRRΔE8) suppressed the function of SIGIRR. In mice that expressed SIGIRRN86/102S, the severity of inflammatory CRC was increased 16. In addition, IL-1R could be used as an indicator. Patients with CRC who did not respond to CTX therapy had higher levels of IL1-R1. Moreover, for patients with consensus molecular subtype 1 (CMS1) CRC and CC subtypes 3 (CCS3), IL-1R1 can be used as a predictor of patient survival 16. Increased IL-1R2 enhanced the expression of angiogenic factors in CC, hence, IL-1R2 may be used as a prognostic marker 17.

#### IL-1β

The role of IL-1β in CRC is closely related to the NF-κB pathway. Through the NF-κB pathway, IL-1β increased the expression of miR-181a, thereby inhibiting phosphatase and tensin homologue (PTEN) expression and promoting the proliferation of CC cells 6. In another study, IL-1β increased the expression of MiR301A in intestinal epithelial cells (IECs) in colitis-associated cancer (CAC) patients, thus inhibiting BTG anti-proliferation factor 1 (BTG1) expression and subsequently promoting the IL-1β-related NF-κB pathway 18. Therefore, blocking the NF-κB pathway is an idea for the future treatment of CRC. Oroxylin A 19, GEN-27 20, tauroursodeoxycholic acid (TUDCA) 21 and an ethanol extract obtained from the aerial parts of Artemisia princeps Pampanini cv. Sajabal (EAPP) have been verified as a blocker of the NF-κB pathway, which would inhibit CRC. In addition, EAPP can inhibit tumour growth by downregulating the expression of pro-inflammatory factors including IL-1β and decreasing the level of anti-apoptotic proteins such as XIAP, cFLIP, survivin, and Bcl-2 22.

**Figure 1:**
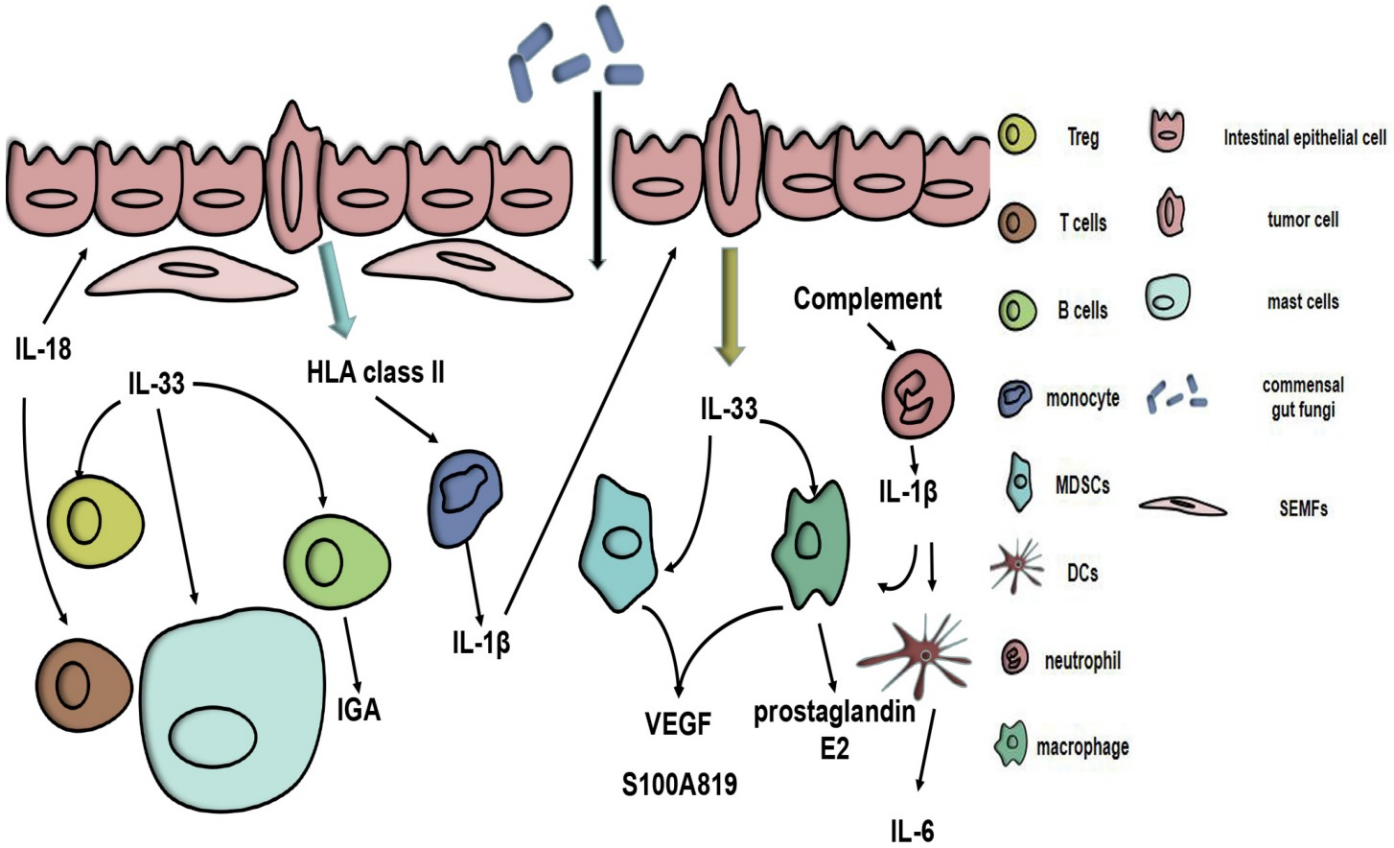
** Potential role of the IL-1 family in colorectal cancer.** HLA class II antigens expressed on CRC cells stimulate the resting monocytes to produce IL-1β. Complement immunity triggers neutrophils to release IL-1β, which can cause myeloid cells to produce IL-6 and increase the IL-17A response. IL-1β can act on IECs and CRC cells. IL-33 can be secreted by vascular endothelial cells and tumour cells. Tumour-secreted IL-33 can act on myeloid cells, causing them to release S100A8/9 and VEGF, thereby altering the tumour microenvironment. IL-33 can also trigger macrophage production of prostaglandin E2, alter the phenotype of Tregs and activate SEMFs and mast cells. The production of IGA in B cells is triggered by IL-33. IL-18 can stimulate epithelial restitution and activate the T cell response, thus enhancing the integrity of the intestinal barrier.

The tumour microenvironment (TME) is of vital importance during the development of tumours. In CRC, the TME also affects the genesis and development of CRC, and some of these processes are related to IL-1β. Compared with normal tissue, a high expression of IL-1β was connected with CRC intestinal mucosa and was also correlated with high expression of HLA class II molecules. Moreover, HLA class II triggers monocyte activation, hence increasing IL-1β expression 23. In addition, LPS can trigger the expression of IL-1β in neutrophils. In the CAC milieu, IL-1β produced by neutrophils can induce intestinal mononuclear phagocytes (MPs) to produce IL-6, thereby promoting tumour formation 24. A complement/neutrophil/ IL-1β-myeloid cells/IL-17A axis is a recently proposed model that explains the impression of the complement system on CRC 5. All the evidence suggests that, because of the relationship between the TME and IL-1β, changing the TME may be a clinical strategy (Fig. 1). Resveratrol, which is a natural polyphenolic compound found mainly in grapes and their seeds, can alter the immune equilibrium between peripheral blood mononuclear cells (PBMCs) and CRC cells 25. In addition, as a chemotherapeutic agent, CPT-11 has been used to treat a wide range of cancers, including CRC. However, its toxic effects limit its use. CPT-11 can cause neutrophil infiltration via the IL-33/ST2 axis, promote macrophage infiltration in intestinal tissues and activate the NLRP3 inflammasome, causing a surge in IL-1β, which then leads to corresponding side symptoms 26, 27. Fortunately, andrographolide (Andro) inhibits the NLRP3 inflammasome in macrophages *in vivo* and *in vitro*, which reduces IL-1β production and thereby reduces the risk of CAC 28.

### Members with tumour-suppressive effects

#### IL-18

Inflammasome-IL-18 axis can act on natural killer (NK) cells to express fasL, thereby having a tumour-inhibiting effect 29. Mutations in the Mediterranean fever gene (Pyrin-IL-18) axis can improve intestinal barrier integrity and prevent CRC formation 30. The interaction of the intestinal flora with ILs has been an innovative thought for CRC treatment. A latest study shows that the commensal fungi-SYK- CARD9-IL-18 axis can prevent CC 31. Since IL-18 can induce T cells to secrete IFN-γ, the specificity of IFN-γ in tumours is still confusing. Furthermore, in a mammalian CRC model, a plasmid vector constructed with the human telomerase reverse transcriptase (hTERT) promoter, IL-18 and herpes simplex virus thymidine kinase (HSV-TK) had a good therapeutic effect 32.

#### IL-36 & IL-37

The studies of IL-36 and IL-37 are limited. IL-37 was found to inhibit the development of CC cells by inhibiting β-catenin, which implies an inhibitory effect of IL-37 on CRC 33. In human CRC, IL-36γ produced by vasculature cells is associated with the conservation of the tertiary lymphoid structure (TLS). Macrophage-produced IL-36Rα is related to immunosuppressive markers, including PD-1, CTLA4 and PD-L1 immune-checkpoint markers 34. In CRC specimens, reduced disease-free survival (DFS) and overall survival (OS) were closely related to low level of IL-37 and high numbers of CD66b+ neutrophil, suggesting that intratumoural IL-37 and CD66b+ neutrophils can be used as independent factors of clinical prognosis for CRC patients 35.

### Members with both tumour-promoting and tumour-suppressive effects

#### IL-33

IL-33 can alter the TME by recruiting CD11b+GR1+, CD11b+F4/80+myeloid cells and macrophages 7, 36, activating subepithelial myofibroblasts (SEMFs) and mast cells 37, and altering the phenotype of Tregs 11, thus promoting the progression of CRC. In addition, IL-33 can affect endothelial cells to promote angiogenesis 7 and activate NANOG, NOTCH3 and OCT3/4 to enhance CC cell stemness 36. sST2 can inhibit M2a polarization, macrophage infiltration, and Th1 and T helper 2 (Th2) responses, thereby promoting CRC 12. A latest research found that the expression of FoxP3 mRNAs was increased in CRC tissues. IHC analysis found that sST2 was correlated with Treg cells which actively express foxp3, implying that sST2 may increase Treg cells to change the TME to make CRC more likely to develop. Additionally, sST2 was interrelated with the poor prognosis of CRC patients 38.

As ILs research progressed, it was discovered that IL-33 could suppress CRC as well. By activating NF-κB in mesenchymal cells and inducing IFNγ gene expression in non-haematopoietic cells, IL-33 can suppress sporadic CC because induced inflammatory factors can exert antitumour effects 39. In addition, in IL-33-deficient mice, the risk of CAC increased due to an increase in IL-1α, while IL-33 maintained intestinal microbiota homeostasis by triggering the production of IGA in B cells, thereby inhibiting IL-1α-induced CAC 40. ST2 can reduce Treg infiltration and increase CD8+ T cells, thereby inhibiting CRC 11.

## IL-2 Family

First described as T cell growth factor in 1976, IL-2 was found in the supernatant of mitogen-activated human T cells, where it played an essential role in supporting the growth and proliferation of T cells *in vitro* 41. The basis for ensuring these biological functions is the combination of IL-2 with its receptors, which are composed of 3 subunits (IL-Rα, IL-2Rβ, and IL-2Rγ). Among them, IL-2Rγ was found not only in IL-2, but also in IL-4, IL-7, IL-9, IL-15 and IL-21. Subsequently, these cytokines were classified into the same family, the IL-2 family (also the γ-chain family or γc family) 42. As has been reported recently, IL-2, IL-9 and IL-15 were elucidated to exert antitumour effect and were regarded as promising CRC treatment candidates 44-46, 58. On the contrary, it was demonstrated that IL-4 and IL-7 were significantly overexpressed in CRC tissues and played negative roles in CRC progression 49-52. Moreover, the function of IL-21 on CRC progression is controversial, with both tumour-promoting and antitumour effect have been reported 47, 48, 59-61.

### Members with tumour-promoting effects

#### IL-4

IL-4 is produced by Th2 cells and has a wide range of effects on T cells, B cells, etc 53. The association between IL-4 and the abnormal STAT6 activation, which mediates signal transduction and promotes metastatic processes of cancer, was recently confirmed in CRC 51. The activation of STAT6 in its transcription is induced by the binding with specificity protein 3 (SP3) in the promoter region of STAT6 gene, which was mediated by the downstream factor of IL-4 named E2F1. Additionally, the overexpression of several epithelial-mesenchymal transition (EMT) drivers, including zinc finger E-box-binding homeobox (Zeb) 1 and Zeb2, was also confirmed, indicating that IL-4 promoted tumour progression via E2F1/SP3/STAT6 axis 51.

Rapid tumour growth is caused mainly by abnormal cell proliferation and inhibited apoptosis, and is the basis for tumour proliferation. In recent studies, IL-4 has been reported to be highly involved in this process. IL-4 secretion regulated by the upstream miR-195/NOTCH axis was confirmed to promote rapid tumour growth through activating recruitment and polarization of the M2-like tumour-associated macrophages, which had long been considered as a risk factor for CRC progression 52. In addition, EMT is considered to be positively related to the invasion and metastasis of tumour cells. The participation of IL-4 in EMT has been highlighted recently. As has been reported, exogenous IL-4 stimulation decreased the membranous epithelial marker E-cadherin level and increased cytoplasmic mesenchymal marker vimentin level at the mRNA and protein levels in CRC cell lines, and both E-cadherin and vimentin were biomarkers representing the stimulation of EMT in cancer cells 51. Consistent result was also observed in another study based on the secretion of IL-4 in CRC 52.

#### IL-7

Different from other ILs, IL-7 is produced by non-haematopoietic stromal cells, although dendritic cells (DCs) can also produce a fraction of IL-7 54. IL-7 is a cytokine that enhances T cell proliferation and survival and has been listed as one of the "Top Agents with High Potential for Use in Treating Cancer" in 2007 55. Although IL-7 was detected to be elevated in CRC patients compared with the control group and the expression level was associated with metastatic disease and tumour location 49, 50, little progress has been made on the underlying mechanisms of IL-7-induced aggressiveness of CRC. Therefore, more advances should be made to investigate biological function of IL-7 in CRC and investigate related molecule mechanisms, which may contain the crosstalk between IL-7 and other factors in the immune system.

### Members with tumour-suppressive effects

#### IL-2 & IL-15

IL-2 is considered as an important cytokine that induces T-cell-mediated immune response by activating NK cells, T-cell, and is involved in the development of regulatory T cells 43. Similarly, IL-15 maintains homeostasis and induces activation of NK cells and CD8+ memory T cells 56. It is acknowledged that NK cells are key antitumour primary effectors to eliminate CRC cells without prior immunization, and altered phenotype and dysfunction of NK cells in CRC patients caused the limitation of the immune response and were associated with the low survival rate. Notably, a study revealed that a treatment strategy combining cetuximab, IL-2 and IL-15, stimulated NK cells and improved cytotoxicity, which provides new insights into ILs-based CRC treatment approaches 44.

IL-15 is negatively involved in CRC progression via inhibiting the proliferation and promoting apoptosis of CRC cells. In order to enhance the transfection efficiency of the gene vector that carrying IL-15, researchers developed a novel gene delivery system with a self-assembly method by MPEG-PLA and DOTAP (DMA), denoted as DMA-pIL15, and the transfected CRC cells showed effective high level of IL-15 secretion 46. Moreover, Ki67 staining and TUNEL assay were applied to determine the regulatory role of IL-15 on cell proliferation and apoptosis, and the results showed that lower proliferation rate and higher TUNEL-positive rate (representing a higher apoptosis rate) were shown in DMA-pIL15-treated group in comparison with the control group, which demonstrated that IL-15 exhibited antitumour effect via inhibiting the proliferation and promoting apoptosis of CRC cells. Also of note, for most malignant solid tumours, the formation of large numbers of microvessels constitutes the basis for tumour growth and metastasis. By counting the number of CD31-positive vessels in the field, the overexpression of IL-15 caused by gene vector was confirmed to reduce angiogenesis of CRC, which further suggested the positive effect of IL-15 in the invasion and metastasis of CRC cells 46.

#### IL-9

Produced by Th2 cells, Th9 cells, Th17 cells and regulatory T cells, IL-9 is a regulatory cytokine and is known for promoting the proliferation and growth of mast cell 57. In CRC, it was demonstrated that IL-9 was predominantly produced by bona fide Th9 cells. By binding to IL-9R expressed on CD8+ T cells, IL-9 significantly enhanced the expansion of its targeted cells, thus inhibiting CRC progression. Additionally, the biological effect of IL-9 on the immune response was significantly impaired by PD-1/PD-L1-mediated inhibition, which was regarded as a key oncogenic signaling pathway 45. A recent study also confirmed antitumour effect of IL-9, followed by the evidence that the expression levels of IL-9 and its mRNA in CC tissue specimens are significantly lower than those in adjacent tissues 58. This experimental team also demonstrated that IL-9 overexpression inhibited tumour growth *in vivo* and showed that this effect was strengthened by activating regulatory T cell to have the killing effect on CC cells 58.

### Members with both tumour-promoting and tumour-suppressive effects

#### IL-21

IL-21 is produced by NK cells, CD4+ T cells and TH17 cells, and can act on all lymphocyte subsets, DCs and smaller monocytes to enhance the intensity of the immune response 48. It was confusing that both pro-tumour and anti-tumour effect of IL-21 on CRC have been reported. As one of the Th17-derived cytosines, IL-21 was broadly reported to be an essential proinflammatory mediator and promoted CRC progression 59-61. Additionally, the elevated expression level of IL-21 was detected in CRC microenvironment, and it was revealed that IL-21 level was negatively correlated with poor survival rate, suggesting its potential role as a prognostic biomarker 47. On the contrary, IL-21 was also reported to exhibit antitumour effects. CD4+CXR5+PD-1-follicular helper T cell was confirmed to facilitate the expansion of CD8+ T cells via the secretion of IL-21, thus enhancing the expression of CD107a and IFN-γ 48. However, similar to IL-9, the antitumour effect could also be suppressed by the activation of PD-1/PD-L1 signaling pathway 48. In a word, further investigations are urgently needed to figure out the regulatory role of IL-21 in CRC as well as the underlying mechanisms.

## IL-6 Family

The IL-6 family comprises IL-6, IL-11, IL-27, IL-31, leukaemia inhibitory factor (LIF), oncostatin M (OSM), ciliary neurotrophic factor (CNTF), cardiotrophin-like cytokine factor 1 (CLCF) and cardiotrophin 1 (CT-1), which all have the similar structure 62-64. The IL-6 family has recently received widespread attention because of its emerging role in various diseases, such as infection, chronic inflammation, autoimmunity and cancer. Initially named B-cell stimulatory factor 2 (BSF-2), IL-6 was officially given the name IL-6 by The New York Academy of Sciences in 1988 65. IL-6 is mainly produced by macrophages, as well as bone marrow-derived myofibroblasts (BMFs), DCs, IECs and myeloid cells 66-69.

### Members with tumour-promoting effects

#### IL-6

Researches show that IL-6 regulates the progression of CRC when combined with gp130 mainly in three signaling pathways, Shp2-Ras-ERK, JAK1/2-STAT3 and PI3K-Akt-mTOR. The factor in common for most of these pathways is STAT3, which plays a dominant role in all of these pathways 66. A recent study also proved that STAT3 activated by IL-6 played a critical role in the fibroblast activation 70. In a latest study on Wu Mei Wan (WMW), which is a traditional Chinese medicine published in Treatise on Febrile Disease, NF-κB/IL6-STAT3 signaling pathway plays an important role. By making AOM/DSS-induced CAC mouse model, WMW shows a great curative effect on CAC. In this study, it is of great focus on NF-κB/IL-6/STAT3 pathway when exploring the tumour suppression mechanism of WMW 71.

In the progression of CRC, the regulation of each stage of the tumour can be accomplished through these molecular pathways. For example, a study proved that the NF-κB-IL6-STAT3 pathway promoted CRC 72. The activation of the IL-6/STAT3/ERK signaling pathway facilitates the angiogenesis, migration and proliferation of CRC. By activating the JAK2/STAT3 pathway, IL-6 induces the EMT in CRC cells through the β-catenin/Wnt signaling pathway 73. The IL-6R/STAT3/MIR34A feedback loop is also necessary for the EMT and metastasis of CRC cells 74. Through the STAT3 pathway, exogenous IL-6 induces the secretion of tumour-derived IL-6 to create a microenvironment that is favourable for the metastasis of CRC cells 75. Another study found that IL-6 contributed to the environment described above by controlling the secretion of mucin 74. In an up-to-date study, IL-6 has been shown to be involved in the drug resistance of CRC under hypoxia 76. The relationship between IL-6 and other cytokines has been extensively studied. For example, IL-6 enhances tumour angiogenesis in CC through the expression of VEGF 77. At the genetic level, pleiotropic IL-6 secreted by senescent cells has the effect of promoting mitosis 78, and through the STAT3/Gp130 pathway, IL-6 promotes the expression of downstream genes covered by cyclin D1, c-myc, bcl-XL, survivin, etc. 79.

#### IL-11

Similar to IL-6R, IL-11 receptor is a member of the gp-130 dependent receptor group 80. IL-11 plays its part in CRC principally by the similar pathways, like JAK/STAT signaling pathway 81. A previous study shows that IL-11 can promote the migration and proliferation of CRC cells by activating PI3K and P44/P42 MAPK pathways 82. In recent years, there have been few studies concentrate on the effect of IL-11 on CRC. In recent studies, IL-11 prefers to appear in the gp130-STAT3 pathway together with IL-6 as a drug or clinical therapy target. For example, a latest study shows that Bazedoxifene can suppress the effect of IL-11 by inhibiting the phosphorylation of STAT3 and nuclear translocation in CRC 79.

In summary, researchers are more interested in IL-6 than IL-11. IL-6 promotes the growth, angiogenesis, proliferation, migration and formation of the microenvironment on CRC through different pathways. Consequently, the level of IL-6 is also involved in the cytokine profile to show the progress and treatment effect on CC 83. Recent studies on IL-6 have mainly focused on the EMT, metastasis and TME, which contributes to the treatment of CRC. For example, nonsteroidal anti-inflammatory drugs and metformin were used to inhibit IL-6-mediated EMT in CRC, showing a good result in CRC regression 84-86. As an interleukin which has been thoroughly researched in a long time, IL-6 deserves more attention to drug tests and clinical treatment. As cytokines which have similar receptors and pathways, IL-11 may deserve more separate researches to find more inspiration on the treatment of CRC.

## IL-8 Chemokine Family

IL-8 (CXCL8), as a member of the CXC family, is mainly induced by pro-inflammatory cytokines such as IL-1β and TNFα to recruit and activate neutrophils and granulocytes to the inflammation place. IL-8 has an affinity for two types of receptors: CXCR1 and CXCR2. Ligation of IL-8 with different receptors causes different biological outcomes.

### Members with tumour-promoting effects

#### IL-8

Three signaling pathways have been described for IL-8: a major pathway through the activation of intracellular signaling PI3K that induces phosphorylation of its substrate, Akt, which plays an essential role in regulating cell survival, migration and angiogenesis. Other pathways include the MAPK signaling cascade and migration- associated PLC-dependent PKC signaling pathway. In addition, activation of the non-receptor tyrosine kinases Src and FAK contributes to IL-8-mediated cancer cell proliferation, survival and chemoresistance, and Rho-GTPases are also involved in IL-8-induced cancer cell mobility and invasion 87. In recent years, researchers have gradually revealed the exact function of IL-8 against CRC, showing the great progress made in the treatment of CRC patients.

IL-8 is a significant chemotactic stimulus that influences the growth and invasion of CRC cells through different mechanisms. High levels of IL-8 in CRC tissue are correlated with higher tumour grade and increased invasion into the liver. In addition, the level of IL-8 also benefits CRC diagnosis and high level of IL-8 in serum contributes to CRC growth and progression, indicating as a potential biomarker for CRC prognosis 88-90. IL-8 participates in most phases of tumour development from cell proliferation and angiogenesis to cancer metastasis, moreover, some evidence has shown that IL-8 is associated with chemotherapeutic responses 91-93.

IL-8 is involved in CRC growth. Ligation of IL-8 to CXCR1 leads to CRC cell proliferation and angiogenesis. In a CRC murine model inoculated with CRC stem cell (CCSC) *in vitro*, cancer cells deficient in IL-8 or CXCR1 showed decreased proliferation and angiogenesis in CCSC. Recent evidence shows the dysregulated expression of cell-cycle proteins, with a reduction in cyclin D and cyclin B and upregulation of the CDK inhibitory protein P21, was the underlying mechanism for IL-8 effects 94. Similarly, in another study, incubation with a CXCR2 neutralizing antibody in HCT116 colorectal carcinoma cells inhibited cell proliferation. Moreover, in this study, the HCT116 cells lost chemoresistance to 5-fluorouracil (5-FU) 91. 5-FU is a major CRC drug that has shown poor outcomes in patients in recent years due to chemotherapeutic resistance. Further investigations found that IL-8 mediated the chemoresistance due to modulating multidrug resistant 1 (MDR1) via IKK-β/p65 signaling within CRC cells treated with doxorubicin (another clinical drug for CRC) 93. In a CRC cell model induced by HT-29 CRC cell conditioned medium, IL-8 was highly elevated and the conditioned medium induced angiogenesis was attenuated after IL-8 was neutralized, indicating that IL-8 plays a role in CRC angiogenesis. Notably, researchers found that the expression of IL-8 was decreased when curcumin or (-)-epigallocatechin-3-gallate were added, both of which are natural components found in plants 95.

Additionally, IL-8 was also found to play a central role in CRC metastasis by inducing the EMT or resistance to anoikis. A protein-protein network analysis demonstrated that IL-8 was one of the four hub genes associated with CRC metastasis 96. Further investigations showed that IL-8 was significantly higher in patients with stage T3 or T4 CRC, lymph node metastasis or liver metastasis. Treatment of the colorectal carcinoma cell line SW480 with IL-8 and IL-20 combined was shown to induce the expression of an EMT phenotype, by triggering the PI3K/AKT-ERK1/2 cross-talk signaling pathway, while treatments with IL-8 or IL-20 alone induced only an EMT-like phenotype, which suggested that IL-8 influenced but did not drive CRC metastasis 97. In another study, IL-8 was correlated with anoikis resistance by ERK and AKT activation and TOPK upregulation, which consequently enhanced CRC metastasis. The addition of IL-8 to CRC cells decreased the apoptosis rate and increased Bcl-2 expression, which is an important apoptosis suppressor factor. Interestingly, IL-8 only decreased apoptosis when the cells cultured in suspension lost cell-cell adhesion yet not under cell attachment conditions. In the same case, further investigation demonstrated that the IL-8 downstream pathways of PI3K/AKT and ERK were involved in an anti-anoikis effect. In addition, elevated TOPK expression under IL-8 treatment was shown to be inhibited by the AKT inhibitor MK2206, suggesting that TOPK may be the downstream signaling target of AKT during anti-anoikis action 89. The elevated level of IL-8 was shown to upregulate integrin αvβ6 in a dose-dependent manner, which consequently contributed to liver metastasis via the ERK and Ets-1 signaling pathways. Integrin αvβ6 plays a vital role in the proliferation, apoptosis, metastasis and matrix metalloproteinase secretion of CRC, and human CRC cells with silenced αvβ6 show a reduction in IL-8-induced migration 98. Besides, recent studies reported that IL-8 was required for the expression of cancer stem cell (CSC) properties through protein O-GlcNAcylation promotion, glucose uptake stimulation and glucose transporter 3 (GLUT3) and glucosamine fructose-6-phosphate aminotransferase (GFAT) upregulation 99.

**Figure 2:**
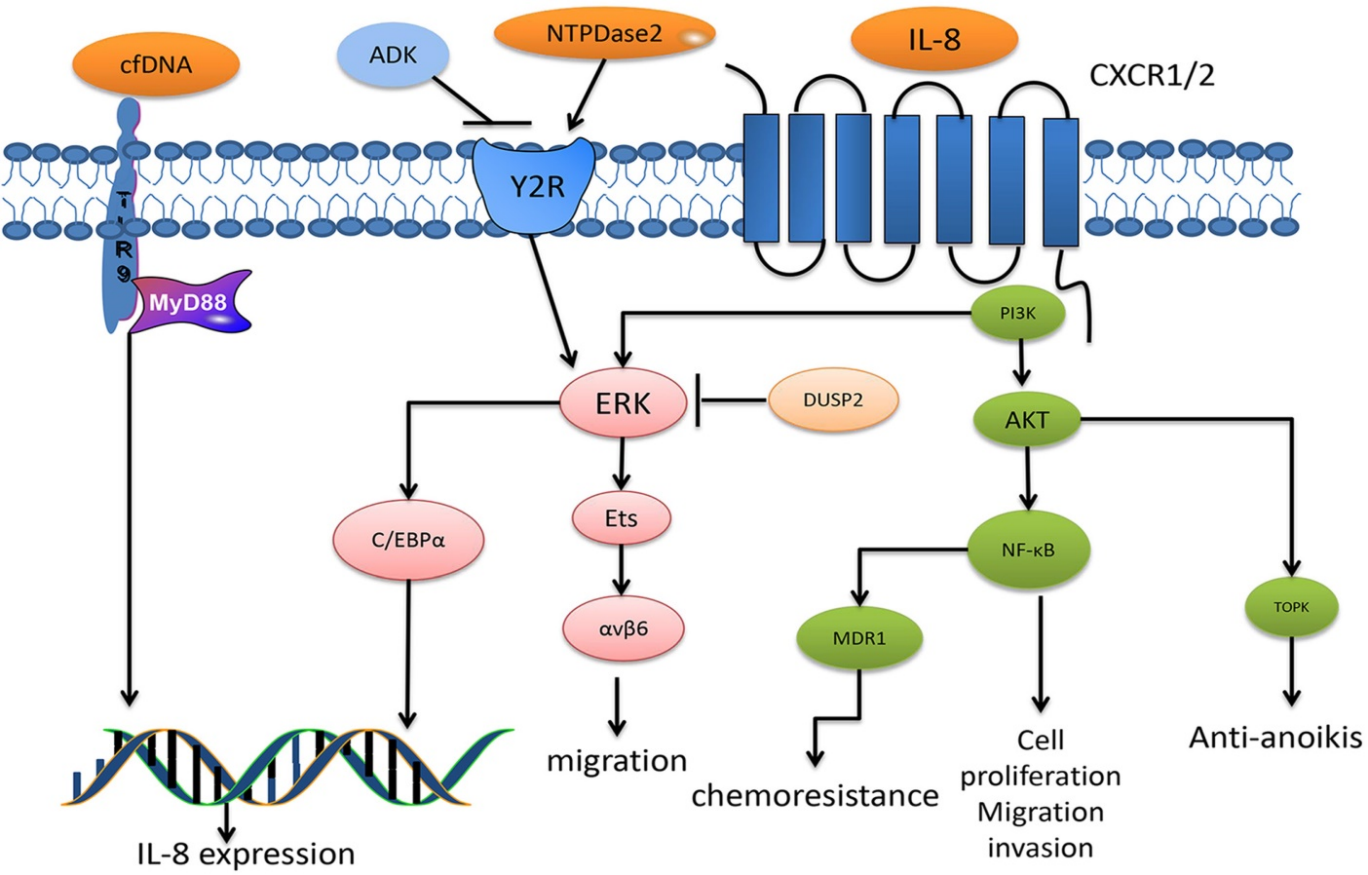
The diagram summarizes the major signaling pathways of IL-8 in CRC. IL-8 binds to G protein-coupled receptor CXCR1 or CXCR2, leading to the activation of PI3K, which induces the phosphorylation of Akt and ERK, respectively. The Akt signals have been reported to activate transcription factors NF-κB, which is associated with CRC cell proliferation, migration and invasion, and is particularly correlated with chemoresistance after MDR1 activation. In addition, TOPK, reported a downstream factor of Akt, mediates anti-anoikis. Another ERK induces the phosphorylation of Ets transcription factors, thus upregulating integrin αβv6 expression, which mediates CRC migration. In addition, ERK signaling also activates C/EBPα, thus promoting IL-8 expression. DUSP2 is a termination factor of ERK activation, and studies have reported its inhibitory role against ERK in CRC. Cell-free DNA activates TLR9-MyD88 signaling, thus promoting IL-8 expression in CRC. NTPase2 binds to Y2R to activate ERK signaling, especially that which leads to mediated IL-8 expression. In addition, the ADK antagonist NTPase2 functions by binding to Y2R.

Studies have also revealed some upstream regulators of IL-8. Several regulators induce IL-8 production, such as SRSF3-TRs, NTPDase2, human neutrophil peptides (HNPs), ring finger domain protein 183 (RNF183) and circulating cell-free DNA (cfDNA), each of which triggers IL-8 expression in terms of signal transduction, activation of intracellular signaling pathways and regulation of gene expression 100-104. For instance, human neutrophil peptides (HNPs) induced IL-8 expression upon the binding of the P2 receptor or P2Y6 and the phosphorylation of components in the ERK1/2 signaling pathway 100. Adenylate kinase (ADK) sustainably activated the P2 receptor signaling pathway, and cfDNA activated TLR9-MyD88 signaling, thereby inducing IL-8 expression 101, 102. Studies also found some regulators that inhibited IL-8 expression, including microRNA-204 (miR-204) and DUSP2 105,106. DUSP2 regulates IL-8 via ERK1/2 signaling and the C/EBPα transcriptional factor. Hypoxia inhibits the expression of DUSP2, leading to the elevation of IL-8, which indicates that the hypoxia-dusp2-IL-8 pathway may be considered a new therapeutic approach 106 (Fig. 2).

Taken together, IL-8 absolutely is a critical cytokine for CRC progression. Researches of IL-8 on CRC metastasis are relatively more mature than other function investigations. According to studies mentioned above, immunotherapy combining IL-8 and IL-20 may be a nice strategy for inhibiting CRC metastasis. On the other hand, it requires further researches to investigate detailed molecular mechanisms of IL-8 in enhancing angiogenesis and maintaining CSC properties. Surprisingly, the curcumin or (-)-epigallocatechin-3-gallate for decreasing the level of IL-8 in CRC has brightened our eyes, which has potential for developing as a new chemotherapy drug. Though, more researches are required to unveil the underlying mechanisms.

## IL-10 Family

Six direct members are involved in IL-10 family: IL-10, IL-20 subfamily members IL-19, IL-20, IL-22, IL-24, IL-26, and a distant family group of IFNs, containing IL-28A, IL-28B and IL-29 107. Studies have shown that two family members (IL-10 and IL-22) are closely related to CRC, suggesting that they are potential therapeutic prospects. IL-10 was first discovered to be secreted by Th2 cells in mice in 1989, and since it inhibited the synthesis of IL-2 and IFN-γ, it was primarily represented as secreted cytokine synthesis inhibitory factor (CSIF). IL-22 is another essential cytokine in CRC. First discovered as IL-10-related T cell-derived inducible factor (IL-TIF), it can be produced by most of the lymphocyte subsets, which mainly are ILC3, Th17 and Th22 107-110.

### Members with tumour-promoting effects

#### IL-22

IL-22 exerts its biological effects by binding to its heterodimer receptor complex which consists of two subunits, IL-10R2-specific and IL-22-specific receptor IL-22. In addition to its functional receptor, an endogenous antagonist, IL-22-binding protein (IL-22BP, known as IL-22RA2) is a natural agent to inhibit IL-22 bioactivity 111. The primary downstream signaling targets of IL-22 are STAT3, STAT1 and STAT5, which are activated by the phosphorylation of Jak1 and Tyk2. Moreover, IL-22 also induces the activation of the MAPK, NF-κB and PI3K-Akt-mTOR signaling pathways.

As IL-22 influences gut homeostasis, it is intimately involved in CRC. In colorectal tissue, IL-22 is a significant tumour-promoting cytokine influencing tumorigenesis, stemness, anti-apoptosis and cell proliferation. High levels of Th22 and IL-22RA1 in a colorectal site are associated with CRC. The increase in Th22 represents a shift from early stage tumour progression to the advanced stage, and a reduction in IL-22RA1 gene expression correlates with low differentiated CRC grade and worse patient outcomes 112,113.

The role of IL-22 in the process of carcinogenesis may be attributed to DNA damage induced by synergism with IFN-γ or helicobacter hepaticus (Hh) infection to produce nitrogen oxide intermediates (iNOS). As reported previously, in a RAG-deficient murine model of carcinogenesis induced by Hh, the iNOS and NO expression was very low in IL-22-deficient mice. However, tumour development was more rapid, and the degree of DNA damage was higher in the control group. Another study showed that IL-22 potently synergized with IFN-γ for iNOS expression via STAT3 activation in human DLD-1 colon carcinoma cells 114,115.

IL-22 is also involved in CRC progression. It is involved in cell proliferation, differentiation, apoptosis and invasion through STAT3, NF-κB, ERK1/2 and so on 116,117. Among them, STAT3 plays a dominant role. Treated with IL-22, RKO colorectal carcinoma cells showed enhanced cell proliferation and reduction in apoptosis, whereas these effects could be eliminated after the addition of STAT3 inhibitor S3I-201 118. Further studies indicated that STAT3 can bind to the DMBT1 promoter region, thereby promoting tumour progression. DMBT1 was originally identified as a tumour suppressor gene in medulloblastoma, whereas accumulating evidence confirms that DMBT1 produces effects in innate immunity and epithelial cell differentiation and binds to viral or bacterial pathogens. In this study, DMBT1 was shown to be upregulated in SW403 CRC cells treated with IL-22 116. These studies indicate that IL-22 promotes the proliferation and inhibits apoptosis of cancer cells via the STAT3 signaling pathway.

IL-22 has been regarded as a regulator of CCSC self-renewal and expansion. Targeting IL-22 activation in CCSC-induced CC with IL-22 antibody dramatically reduced primarily tumour volume, delayed tumour development and increased mouse survival. The results inspired the authors to investigate the mechanisms undergirding these findings, and they discovered that IL-22-mediated tumour stemness was associated with the methylation of H3K79 in three core cancer stemness genes: NANOG, SOX2 and Pou5F1. Their investigation also proved that p-STAT3 can bind to the disruptor of the telomeric silencing 1-like (DOT1L) promoter region and thus induce DOT1L overexpression, which plays a key role in the methylation of H3K79 119.

Previous studies reported that IL-22BP played a crucial role in controlling the expression of IL-22. Infected with an IL-22BP overexpression vector in C26 CC cells, the effects of tumour angiogenesis and anti-apoptosis were attenuated, and the expression of p-STAT3, VEGF-A, Bcl-xL and survirin was downregulated, which provided a theoretical basis for the use of IL-22BP in the treatment of CRC patients 120.

**Figure 3:**
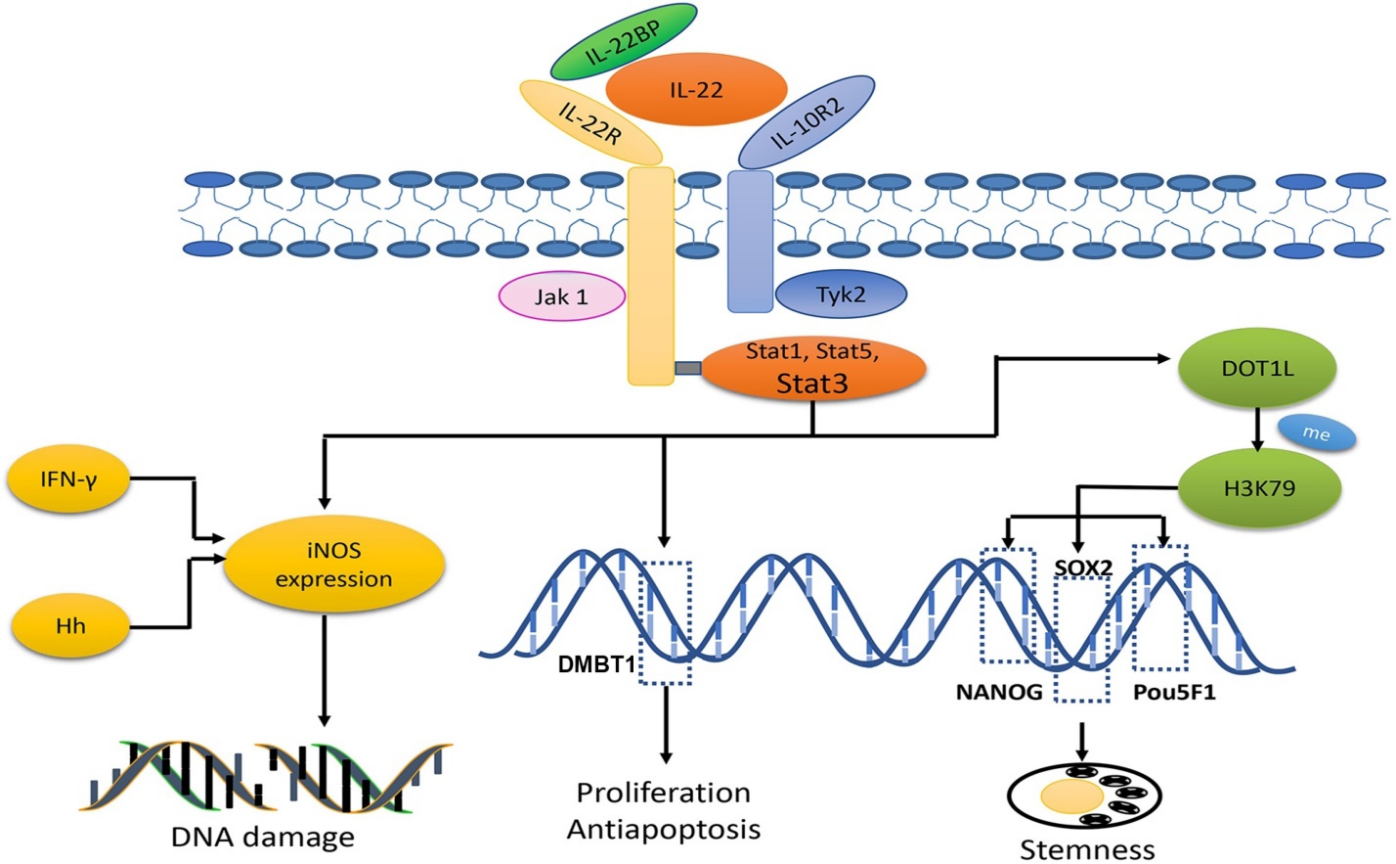
IL-22 signaling is transduced through heterodimer receptor complexes composed of IL-10R2 and IL-22. In addition, there is an endogenous antagonist, IL-22BP. The primary downstream signaling targets of IL-22 are STAT3, STAT1 and STAT5, which are activated by the phosphorylation of Jak1 and Tyk2. The role of IL-22 in the process of tumorigenesis is attributed to DNA damage induction through its synergistic interaction with IFN-γ or Hh infection to produce nitrogen oxide intermediates (iNOS). Furthermore, STAT3 can bind to the DMBT1 promoter region, thereby promoting tumour progression. In addition, IL-22 mediates tumour stemness, which is associated with methylation of H3K79 at three core cancer stemness genes, NANOG, SOX2 and Pou5F1.

In summary, IL-22 has dominant performance in CRC carcinogenesis and stemness, pointing that IL-22 identified as an early stage tumour target could be a proper thinking. Though IL-22 relevant cells and receptors are represented as biomarkers for CRC prognosis and diagnosis, there's no direct evidence to confirm that IL-22 can be used as a biomarker. IL-22 BP is a natural factor for attenuating IL-22 effects in tumour angiogenesis and anti-apoptosis, which showing a prospective potential for CRC treatment (Fig. 3).

### Members with both tumour-promoting and tumour-suppressive effects

#### IL-10

With a high binding affinity for IL-10RA and IL-10RB heterodimer 121, 122, IL-10 conducts to the recruitment and activation of JAK to initiate the downstream signaling pathway and transcription factors (STAT3, STAT1 and STAT5). Among them, STAT3 plays a dominant role in IL-10 signaling.

IL-10 is a crucial immunosuppression agent, and the lack of IL-10R in colorectal tissue could cause severe spontaneous colitis, which poses a risk for CRC initiation 123. However, the role of IL-10 in cancer pathogenesis and development is complex. In combination with CY, lentivectors encoding shRNA specific to IL-10 (shIL-10 LVs) silenced IL-10 expression and inhibited CRC growth. Moreover, the author discovered that IL-10 deficiency enhanced the efficacy of DC-based immunotherapy, reduced MDSC and Treg levels in the TME and promoted Th1-type antitumour responses, indicating that IL-10 plays a tumour-promoting role in CRC 124. However, in another study, a murine tumour model treated with genetically modified lactic acid bacteria (GM-LAB) and engineered to produce IL-10 or antioxidant enzymes showed CC tumour inhibition 125, indicating that IL-10 might suppress tumour growth. The expression level of IL-10 was found lower in patients 7 days after CRC surgery than before, and patients with recurrence CRC after the surgery had significantly higher level of IL-10, indicating that IL-10 can be a prognostic biomarker in CRC 126. Anyway, whether IL-10 is a tumour promoting agent or inhibitor still needs further studies to elucidate.

## IL-17 Family

The IL-17 family consists of 6 cytokines which are structurally related, IL-17A (also called IL-17), IL-17B, IL-17C, IL-17D, IL-17E (also called IL-25) and IL-17F 127. These family members perform distinct functions. For example, IL-17F is involved in mucosal host defence; IL-17E was confirmed to be an amplifier of TH2 immune responses, and IL-17A shows the highest involvement in tumour progression (including CRC), as well as inflammation and autoimmunity 128. In previous studies on these cytokines, IL-17A and IL-17F catch our eyes. While IL-17A increased tumour growth and metastasis, the IL-17F has anti-tumour effects on CRC 61.

### Members with tumour-promoting effects

#### IL-17A

**Figure 4:**
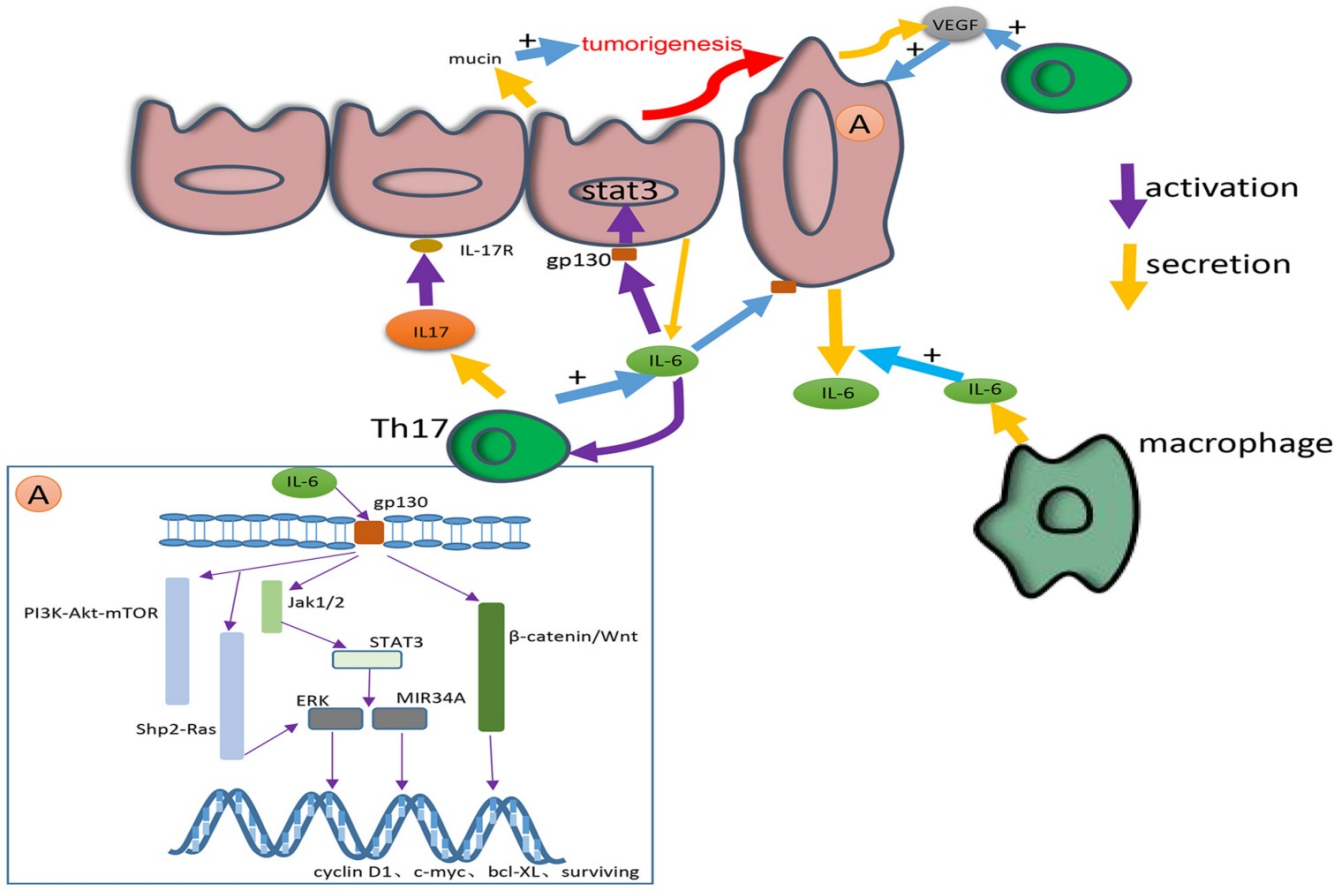
This picture shows the secretion, signaling pathways and cross-talk of IL-17 and IL-6. IL-6 is mainly secreted by macrophages in epithelium and colorectal cancer tissue. IL-17 is mainly secreted by Th17. IL-17 can promote the secretion of IL-6. In addition, IL-6 can activate Th17. The IL-6 secreted by macrophages can induce the secretion of tumour-derived IL-6. IL-6 exerts its effect by combining with gp130 to activate various pathways, including the IL-6/STAT3/ERK, JAK1/2-STAT3, β-catenin/Wnt, PI3K-Akt-mTOR and Shp2-Ras-ERK pathways. Through these pathways IL-6 can change the expression of tumour cell genes such as cyclin D1, c-myc, bcl-XL, survivin, etc., to great effect. IL-6 can also facilitate the secretion of mucin and promote the tumorigenesis. IL-17 consisting of IL-17R facilitates the secretion of VEGF and promotes tumorigenesis and angiogenesis. Both IL-6 and IL-17 contribute to a microenvironment that is favourable for the growth of colorectal cancer.

IL-17A is involved in the growth, angiogenesis and metastasis of CRC. IL-17A indirectly promotes the pathogenesis and development of CRC by inducing the secretion of IL-6 through the STAT3 pathway 129. Additionally, IL-17A increases the levels of cytokines and chemokines produced by myeloid cells to change the tissue environment and microbiota in CRC 130. A previous study found that IL-17A in CRC cells increased the expression of Sca-1 131, which facilitated cell cycle progression and promoted the tumorigenesis of CRC. Through IL-17-STEAP4-XIAP axis, CC can be promoted by inducing copper uptake in the inflammatory response 132. Moreover, by binding to its receptor on vascular endothelial cells, IL-17A promotes the secretion of VEGF, thus inducing tumour angiogenesis 133 (Fig. 4).

### Members with tumour-suppressive effects

#### IL-17F

In a previous study, we found that compared with the expression in normal human colon epithelial cell, IL-17F was observably decreased in CC tissue. In this study, IL-17F was also proved to have the tumour suppression effect in CC possibly by inhibiting tumour angiogenesis 134.

In summary, we speculate that IL-17 has great potential for the treatment of CRC and a study shows that the overexpression of IL-17A may be closely related to the poor prognosis in patients with CRC 135. Besides, the underlying molecule mechanisms on IL-17A and basic function on IL-17F remain to be further studied before more effective clinical therapies and drugs are found.

## Clinical Application

Since the role of ILs in CRC has been extensively studied in the laboratory and the prominent roles of ILs in CRC have been found, the clinical application of ILs in CRC treatment has attracted attention (Table 2).

### Playing the role directly

Injecting ILs with a tumour suppressing effect in CRC patients is a straight and effective manner. A daily dose of pegylated recombinant human IL-10 (AM0010) was injected subcutaneously into the recruited patients with advanced solid tumours, including CRC, renal cell cancer (RCC), etc. Although there was a certain degree of side effects, such as anaemia, fatigue, thrombocytopaenia, fever, and injection site reactions, the side effects remained within an acceptable range. In this phase I study, according to immune-related response criteria, it was confirmed that AM0010 had a good antitumour effect on many solid tumours, including CRC, indicating that it is worthy of more clinical trials in the future 136.

### Targeting ILs in CRC treatment

To improve CRC outcomes in clinical trials, inhibiting ILs that promote CRC became the choice treatment in clinical trials. MABp1, derived based on the human immune response, is a monoclonal antibody against IL-1α. In a phase III trial, the researchers proposed a new and useful evaluation standard: the primary endpoint, which refers to a stable increase in lean body mass (LBM) and stabilization or improvement in at least two signs as determined by the EORTC QLQ-C30 (fatigue, pain, and anorexia). Among those recruited with metastatic CRC, 33% of the patients who received at least one dose of MABp1 and 19% of the patients who received at least one dose of placebo achieved the primary endpoint (*p*=0.0045). The two groups have shown no significant differences in serious adverse events. This finding indicated that MABp1 provided a new strategy for the treatment of advanced CRC 137. HuMax-IL8 (now known as BMS-986253) is a monoclonal antibody with the ability to inhibit IL-8. After receiving HuMax-IL8 treatment, 15 patients with solid tumours, including 4 CRC patients, were safe without severe side effects. At the same time, their serum was tested and a decrease in IL-8 was found. This is the first trial on IL-8 blockade, and clinical trials on BMS-986253 are continuing 138. However, the outcome was different for another drug, siltuximab, which is a monoclonal antibody with a high affinity for IL-6. In a Phase I/II clinical trial, siltuximab had no effect on solid tumours, including Kirsten rat sarcoma-2 (KRAS)-mutant cancers 139. Nevertheless, the findings from this experiment indicated a decrease in p-STAT3 after siltuximab treatment, and the researchers speculated that siltuximab had no clinical effect possibly because of tumour autocrine IL-6 and tumour heterogeneity.

### Using in adjuvant therapy

As the tumorigenesis and development of CRC are complex, there are many factors that may function in these processes. Therefore, creative ideas for the use of ILs as adjuvant therapies are important. DC-based cancer immunotherapy is an effective treatment for stimulating the immune response against tumours. However, maintaining long-term T cell responses has been problematic. In a phase I clinical study, researchers recruited 12 patients with metastatic CRC, for which conventional treatment had no significant effect. First, carcinoembryonic antigen (CEA)-pulsed DCs and tetanus toxoid were injected into the patients, followed by three injections of CEA-pulsed DCs and a small amount of injected IL-2. The small amount of IL-2 added after the inoculation helped to prolong the T cell response time. This clinical trial study found that this immunotherapy had a certain clinical significance without obvious side effects and deserved to be further researched 140.

### Functioning as clinical indicator

In addition, ILs can be used as clinical indicators for use in some clinical judgements. In two randomized phase III trials, it was confirmed that the IL-6 rs2069837 genotype could be used as a clinical prognostic factor for mCRC patients who received bevacizumab-based chemotherapy 141. For the mCRC treatment, health-related quality of life (HRQoL) is a significant outcome. The researchers tested HRQoL levels of 512 mCRC patients receiving cetuximab and found that patients with high serum IL-6 or C-reactive protein (CRP) had worse HRQoL outcomes than patients with normal levels 142. In another trial, the researchers separated 40 patients with CRC into two groups: using open colectomy as group A and using laparoscopic colectomy as group B. The researchers measured IL-6, TLR-2, TNF-α, TLR-4 and high-sensitivity CRP (hsCRP) levels and concluded that laparoscopic colectomy had short-term clinical advantages compared with open colectomy owing to the higher levels of IL-6, TLR-2, TLR-4 and hsCRP in group A than compared to those in group B 143.

## Conclusion

The role of cytokines in cancer is fascinating. As a significant component of cytokines, ILs have enlightening roles in CRC. In this review, we have summarized the significant roles of various IL families in CRC as reported in recent years. As new members of ILs which may exert enormous effects on CRC are constantly being discovered, the underlying molecule mechanisms of new members on CRC should be given enough attention. In the case of ILs with detailed laboratory research foundation and bioinformation analysis, clinical applications including clinical side effects, efficacy, and combined use need to be investigated. Evidence shows that ILs have a tremendous connection with plentiful cells within the TME, then describing the communication between ILs and individual cells, including immune cells, may have an indelible role in the future. It's interesting that some ILs such as IL-33, IL-21 and IL-10 have exerted dual influence in the CRC. However it is still confusing whether the dual influence is due to experimental limitations or other undiscovered mechanisms. Ingeniously, some intestinal microorganisms can communicate with ILs and ultimately affect CRC. How intestinal microorganisms alter ILs and how to put this into clinical application cannot be missed.

## Figures and Tables

**Table 1:** Summary of the interleukin families and the roles in CRC

Interleukin Family	Cytokine	Receptor	Functional effect in CRC	Expression Patterns	Reference
IL-1 FAMILY	IL-1α	IL-1R1 IL-1R2	promote metastasis and the chemosensitivity	↑	11 14
	IL-1β	IL-1R1 IL-1R2	promote the proliferation of colon cancer cells, promote tumorigenesis, alter the tumour microenvironment	↑	11 25
	IL-1Rα	IL-1R1 IL-1R2	confusing	↑	10
	IL-33	IL-1R4 (ST2)	alter tumour microenvironment, promote angiogenesis, enhance colon cancer cell stemness, suppress sporadic colon cancer, maintain intestinal microbiota homeostasis	↑	7,12, 37-40
	IL-18	IL-1R5 (IL-18Rα IL-18Rβ)	act on NK cells, improve intestinal barrier integrity	↓	11 30
	IL-36α	IL-1R6	antitumour	─ ─	34
	IL-36γ	IL-1R6	promote inflammatory immune infiltrates	─ ─	34
	IL-37	IL-1R5	inhibit the development of colon cancer cells by inhibiting β-catenin	↓	33 35
IL-2 FAMILY	IL-2	IL-2Rα, IL-2Rβ/IL-2Rγ, IL-2Rα/IL-2Rβ/IL-2Rγ	antitumour	─ ─	43 44
	IL-4	TypeⅠ (IL-4Rα/γc) and Type Ⅱ (IL-4Rα/IL-13Rα1)	promote EMT, proliferation, invasion and metastasis	↑	51 52
	IL-7	IL-7R (IL-7Rα/γc)	promote metastasis	↑	49 50
	IL-9	IL-9R (IL-9Rα/γc )	antitumour	↓	45 58
	IL-15	IL-15R (IL-15Rα/IL-15Rβ/γc)	inhibit proliferation and angiogenesis, promote apoptosis	─ ─	46
	IL-21	heterodimers of IL‑21R and γc	confusing	↑	47-48, 59-61
IL-6 FAMILY	IL-6	gp130 IL-6R	promote mitosis, proliferation, metastasis, migration, angiogenesis and make a microenvironment which is good for the metastasis	↑	Knupfer H, Preiss R. International journal of colorectal disease. 2010;25(2):135-40
	IL-11	gp130 IL-11Ra	facilitate the proliferation of CRC	↑	Putoczki TL, et al. Cancer cell. 2013;24(2):257-71
IL-8 FAMILY	IL-8	CXCR1 and CXCR2	promote cell proliferation, angiogenesis, cancer metastasis, chemoresistance, anti-anoikis, maintain CCSC properties	↑	88-94, 96-99
IL-10 FAMILY	IL-10	IL-10RA and IL-10RB	confusing	↑	123-125 Zadka L, et al. Cytokine. 2018; 110: 116-25
	IL-22	IL-10RB and IL-22R /IL-22BP	promote tumorigenesis, stemness, anti-apoptosis and cell proliferation	↑	110-118
IL-17 FAMILY	IL-17a	IL17R	promote cell cycle progression and angiogenesis, facilitate the occurrence and development indirectly and change the tissue environment and microbiota of CRC	↑	127 Razi S, et al. Cytokine. 2019; 116:7-12
	IL-17b	IL17R	promote tumour	↑	Razi S, et al. Cytokine. 2019; 116:7-12
	IL-17c	IL17R	null	─ ─	127
	IL-17e	IL17R	antitumour	─	Razi S, et al. Cytokine. 2019; 116:7-12
	IL-17f	IL17R	tumour suppression effect possibly by inhibiting tumour angiogenesis	↓	134 Razi S, et al. Cytokine. 2019; 116:7-12

↑: upregulated, ↓: downregulated, ─: unchanged, ─ ─: confusing.

**Table 2:** Clinical trials targeting interleukins in CRC

Clinical trial.gov identifier	Targeted molecule	Phases	Status	Type of CRC
NCT01902849	IL-6 IL-10	N/A	Completed	Colorectal Cancer
NCT00072098	IL-12	Phase 1	Terminated	Colorectal Cancer | Metastatic Cancer
NCT00003046	IL-12	Phase 1	Completed	Stage IV Colorectal Cancer
NCT03542799	IL-12	Phase 1	Not yet recruiting	Metastatic Colorectal Cancer
NCT00004074	IL-12	Phase 1	Completed	Metastatic Colorectal Adenocarcinoma | Recurrent Colorectal Carcinoma | Refractory Colorectal Carcinoma | Stage IV Colorectal Cancer
NCT00020267	IL-2	Phase 1	Completed	Colorectal Cancer
NCT01300858	IL-12	Phase 1| Phase 2	Terminated	Metastatic Colorectal Cancer
NCT00019591	IL-2	Phase 1| Phase 2	Completed	Colorectal Cancer
NCT00019591	IL-2	Phase 1| Phase 2	Completed	CRC
NCT00841191	IL-6	Phase 1| Phase 2	Completed	Metastatic Colorectal Cancer
NCT00841191	IL-6	Phase 1| Phase 2	Completed	CRC
ChiCTR1900023583	IL-1	Phase 2	Not yet recruiting	Colorectal Cancer
NCT03823079	IL-11	Phase 2	Not yet recruiting	Recurrent Colorectal Carcinoma
NCT03823079	IL-11	Phase 2	Completed	Recurrent Colon Cancer | Stage III Colon Cancer | Stage IV Colon Cancer
CTR20191045	IL-1R	Phase 2	In the recruitment	Metastasis of Colorectal Cancer
NCT02090101	IL-1β	Phase 2	Completed	CRC
NCT02919644	IL-2	Phase 2	Active, not recruiting	Metastatic Colorectal Cancer
NCT03222089	IL-2	Phase 2	Withdrawn	Colorectal Cancer
NCT03610490	IL-2	Phase 2	Recruiting	Colorectal Neoplasms
NCT00019331	IL-2	Phase 2	Completed	Colorectal Cancer | Colon Cancer
NCT00176761	IL-2	Phase 2	Completed	Metastatic Colorectal Cancer
NCT00176761	IL-2	Phase 2	Terminated	CRC
NCT02919644	IL-2	Phase 2	Active, not recruiting	CRC
NCT03222089	IL-2	Phase 2	Withdrawn	CRC
2014-000550-12	IL-1α	Phase 3	Completed	Symptomatic Colorectal Cancer Patients Refractory to Standard Therapy
EUCTR2005-003458-81-IT	IL-2	Phase 3	Completed	Colorectal Cancer
2005-003458-81	IL-2	Phase 3	Completed	Advanced Colon-Cancer

## Abbreviations

ADK: adenylate kinase
CAC: colitis-associated cancer
CC: colon cancer
CCSC: colorectal cancer stem cell
cfDNA: circulating cell-free DNA
CRC: colorectal cancer
DCs: dendritic cells
DOT1L: disruptor of the telomeric silencing 1-like
EAPP: artemisia princeps Pampanini cv
SajabalEMT: epithelial-mesenchymal transition
Hh: helicobacter hepaticus
HRQoL: health-related quality of life
IECs: intestinal epithelial cells
IFP: interstitial fluid pressure
IL-22BP: IL-22-binding protein
ILs: interleukins
iNOS: nitrogen oxide intermediates
MDR1: multidrug resistant 1
NK cells: natural killer cells
PTF: perfusable tissue fraction
SEMFs: subepithelial myofibroblasts
SIGIRR: single immunoglobulin IL-1 receptor-related molecule
TBF: tumour blood flow
TME: the tumour microenvironment
